# Totally endoscopic robotic repair of coronary sinus atrial septal defect with concomitant tricuspid annuloplasty

**DOI:** 10.1186/s44215-024-00167-1

**Published:** 2024-09-11

**Authors:** Kazuki Noda, Yosuke Takahashi, Akimasa Morisaki, Yoshito Sakon, Kenta Nishiya, Goki Inno, Yukihiro Nishimoto, Munehide Nagao, Toshihiko Shibata

**Affiliations:** Department of Cardiovascular Surgery, Osaka Metropolitan University, 1-4-3, Asahichou, Abenoku, Osaka 545-8585 Japan

**Keywords:** Unroofed coronary sinus, Atrial septal defect, Robotic surgery

## Abstract

**Background:**

The coronary sinus type of atrial septal defect is rare. Standard treatment typically involves intracardiac repair using conventional sternotomy or thoracotomy incisions; however, robotic technology can offer a feasible alternative due to its ability to provide a high-quality surgical view of this anomaly.

**Case presentation:**

A 72-year-old man presented with asymptomatic atrial septal defect. Echocardiography revealed a direct communication between the left atrium and CS with left-to-right shunt flow and a Qp/Qs ratio of 2.1:1. The coronary sinus type of atrial septal defect was indicated for the totally endoscopic robotic repair considering few comorbidities. We present a successful robotic repair of coronary sinus atrial septal defect with concomitant tricuspid annuloplasty via the right atrium, properly identifying the boundary between the mitral annulus and coronary sinus through a high-quality surgical view.

**Conclusion:**

Robotic repair can serve as a viable and therapeutically effective alternative for cases of coronary sinus atrial septal defect with concomitant tricuspid annuloplasty.

**Supplementary Information:**

The online version contains supplementary material available at 10.1186/s44215-024-00167-1.

## Background

Coronary sinus atrial septal defect (CS-ASD) is a rare type of ASD characterized by incomplete atrioventricular fold formation. This anomaly is a part of unroofed CS syndrome, which may result in a partial or complete defect in the roof of the CS with or without a persistent left superior vena cava (PLSVC) [1]. The standard intracardiac repair approach for this anomaly has traditionally been median sternotomy or thoracotomy; however, robotically assisted repair can be a feasible alternative, as reported in some studies [2, 3]. Herein, we present a successful case of totally endoscopic robotic repair of CS-ASD via right atriotomy in an elderly person.

## Case presentation

**Fig. 1:**
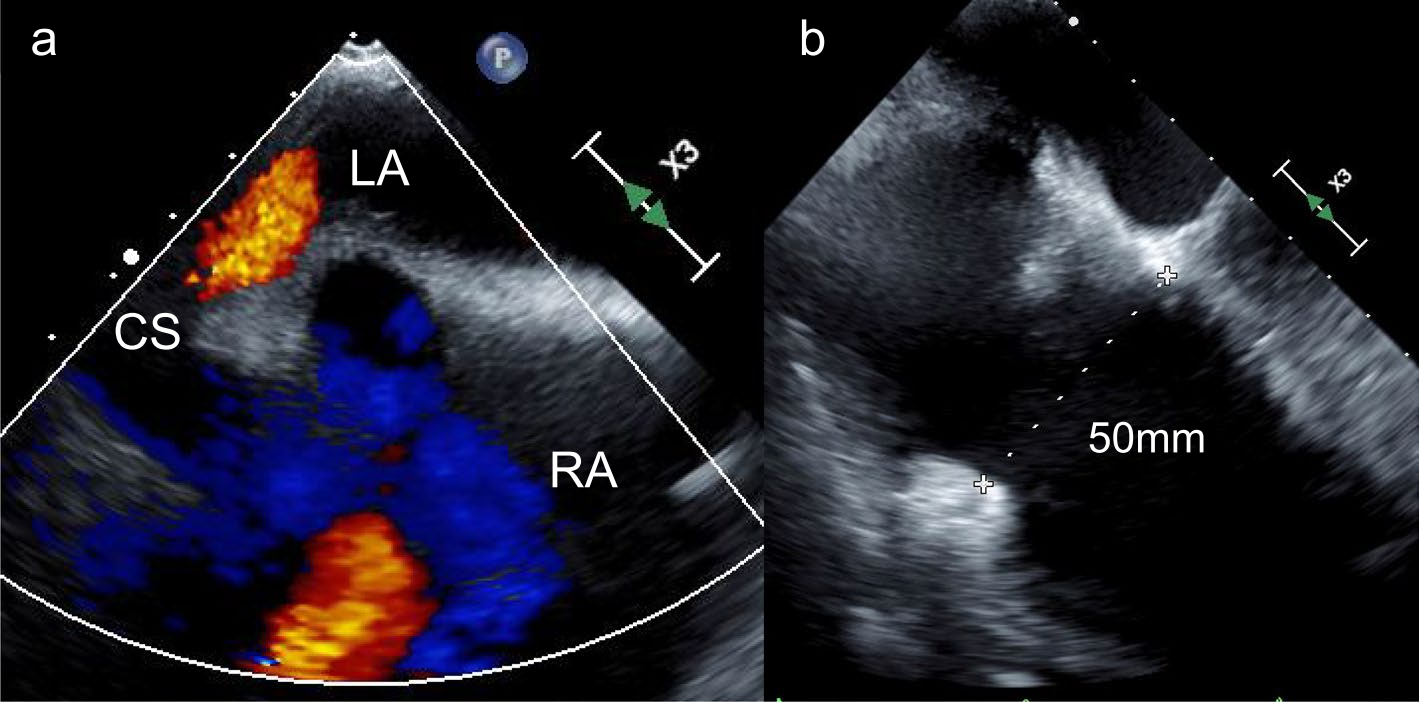
Transesophageal echocardiography revealed a direct communication between the left atrium and CS with left-to-right shunt flow, as observed via color Doppler imaging (**a**). The annulus of tricuspid valve was dilated at end-diastole, 50 mm in diameter (**b**). LA, left atrium; RA, right atrium; CS, coronary sinus

**Fig. 2:**
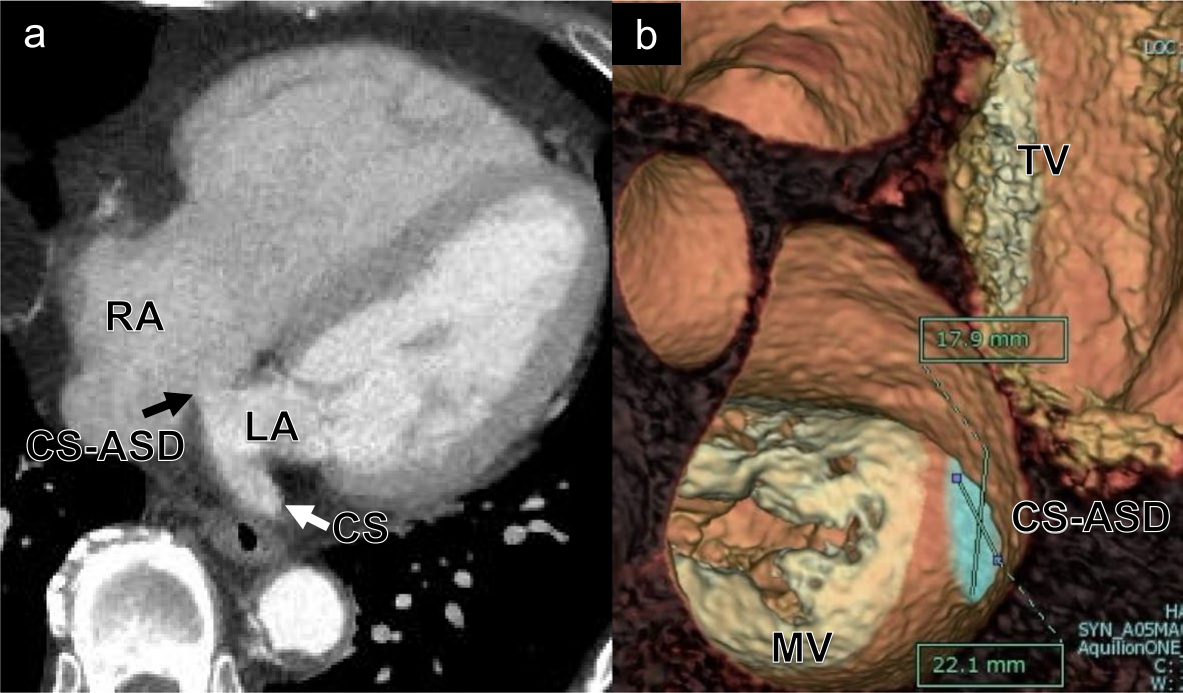
Enhanced CT showed a communication between the left atrium and CS (**a**). 3D reconstruction CT revealed that the defect was located adjacent to the mitral valve annulus, measuring approximately 18 mm × 22 mm (**b**). LA, left atrium; RA, right atrium; CS, coronary sinus; CS-ASD, coronary sinus atrial septal defect; MV, mitral valve; TV, tricuspid valve; CT, computed tomography; 3D, three dimensional

A 72-year-old man with a medical history of hypertension and dyslipidemia was found to have a heart murmur during a periodic health examination. He then visited a local doctor. Transthoracic echocardiography (TTE) revealed an ASD. He was then referred to our hospital. Further evaluation with transesophageal echocardiography (TEE) showed a direct communication between the left atrium and CS with left-to-right shunt flow and a Qp/Qs ratio of 2.1:1, as observed via color Doppler imaging (Fig. 1a). Moreover, mild tricuspid regurgitation with annulus dilatation of 50 mm in diameter was noted (Fig. 1b). Enhanced computed tomography (CT) confirmed the communication between the left atrium and CS (Fig. 2a), measuring approximately 18 mm × 22 mm (Fig. 2b), and excluded any anomalous pulmonary or systemic venous return. Cardiac catheterization revealed a 13% oxygen saturation step-up from the superior vena cava to the right atrium and a Qp/Qs ratio of 1.9:1, and selective coronary angiography confirmed normal coronary arteries. After discussing with the patient, totally endoscopic robotic repair of the defect and concomitant tricuspid annuloplasty, which was indicated for mild tricuspid regurgitation with annulus dilatation over 40mm, was performed due to few comorbidities and optimal anatomical proportions for the robotic approach.

**Fig. 3:**
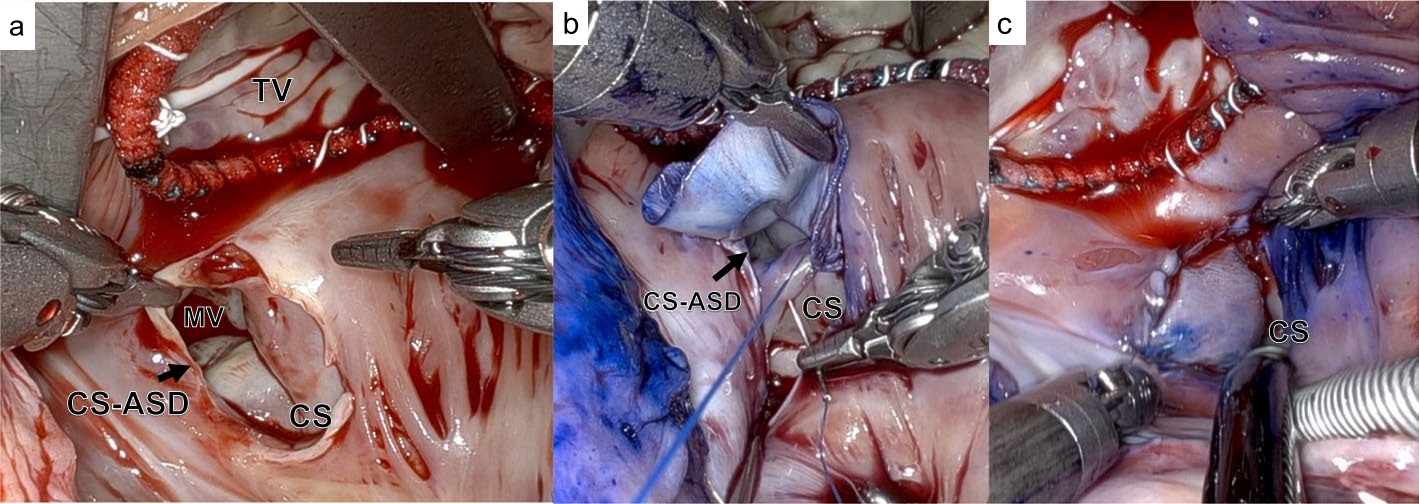
Intraoperative findings showed that **a** the annulus of the mitral posterior leaflet could be confirmed through the defect. **b** Patch repair of the defect was performed with continuous suture, leaving the CS orifice on the right. Finally, there was no residual shunt (**c**). CS-ASD, coronary sinus atrial septal defect; CS, coronary sinus; MV, mitral valve; TV, tricuspid valve

The da Vinci Surgical System Xi (Intuitive Surgical, Inc., Sunnyvale, CA, USA) was used for the procedure. Briefly, the patient was intubated with a double-lumen endotracheal tube, and the right internal jugular vein was cannulated before draping. A 4-cm-long skin incision was made in the third intercostal space which was suitable for approaching CS-ASD and tricuspid valve, guided by preoperative CT. Then, da Vinci ports were inserted in the second for the left arm, third for the retractor arm, and fifth intercostal spaces for the right arm through lateral endoscopic approach using robotics technique [4]. Following systemic heparinization, cardiopulmonary bypass was initiated through the right femoral and subclavian arteries because of the shaggy aorta, and vacuum-assisted venous drainage was performed from the right femoral and internal jugular veins. The da Vinci system was used from the pericardiotomy step. By pulling pericardial traction sutures out of the body, the sufficient surgical space was available to perform the procedures. A venting tube was inserted from the right upper pulmonary vein to the left atrium. Cardiac arrest was induced by direct cross-clamping of the ascending aorta, and tepid blood cardioplegia was infused into the aortic root. After right atriotomy was performed, the femoral venous cannula was repositioned and directly clamping the superior and inferior vena cava by clips. Tricuspid valve and CS-ASD exposure was achieved with the aid of a dynamic atrial retractor controlled from the robotic console. The defect was located at the terminal portion of the CS, which communicated with the left atrium. The defect size was approximately 25 mm × 25 mm, and the annulus of mitral posterior leaflet could be confirmed through the defect (Fig. 3a). Initially, tricuspid annuloplasty using a flexible prosthesis ring was performed with CV4 polytetrafluoroethylene continuous wrapping sutures. Subsequently, after trimming the bovine pericardial patch, patch repair of the defect was performed with 5-0 polypropylene continuous sutures, leaving the CS orifice on the right by the insertion of suction tube (Fig. 3b, c and Additional file 1). Using antegrade blood cardioplegia, CS patency was confirmed by the return of cardioplegia, and simultaneously there was no leakage from the defect under the increased left atrial pressure without venting. After atrial closure in the double layers, the patient was weaned from bypass. The cardiopulmonary bypass and aortic clamping times were 188 and 108 min, respectively.

Postoperative TTE demonstrated no residual shunt. Because the patient had postoperatively episodes of paroxysmal atrial fibrillation, anticoagulation therapy was introduced. He was discharged home at 12 days after surgery and was determined to be clinically well after a year.

## Discussion

CS-ASD is a part of unroofed CS syndrome, which represents an abnormal communication between the CS and left atrium. The morphological types of unroofed CS syndrome have been classified into four groups based on whether the defect is complete or partial and whether it has a PLSVC or not [5]. Herein, the patient had a partially unroofed terminal portion, which was classified as type 4.

Totally endoscopic robotic repair of ASD is safe and feasible [6, 7]. The robotic approach provides not only cosmetic benefits and patient comfort but also a high-quality surgical view and precise suture/cut handling, which are more suitable for complex intracardiac repairs in adults than conventional sternotomy or thoracotomy without robotic techniques. Also, compared to other approaches, totally endoscopic robotic surgery provides quicker postoperative recover thanks to limited skin incision. Conversely, conventional sternotomy provides the comprehensive access and direct visualization that allowing extensive repairs and dealing with unexpected complications. Thus, the approach should be carefully considered taking each advantage into account.

Surgical correction of unroofed CS syndrome is determined by the presence of PLSVC. In cases of unroofed CS syndrome with PLSVC, either direct ligation of PLSVC or rerouting to the right atrium is required [8, 9]. In the absence of PLSVC, as in the present case, defect correction by reroofing the CS or closure of the CS ostium with a patch, if the defect is adjacent to the CS ostium, is indicated [10]. Although repairing an unroofed CS may be challenging, a robotic approach can facilitate the procedure by providing clear visualization of the anatomy and allowing accurate suturing. The key point in this case is accurately identifying the boundary between the mitral annulus and CS. Visualization of the mitral valve annulus and CS roof defect with a zero-degree scope via the right atrium helped close the defect and preserve the CS orifice. To enhance the safety of this procedure, preoperatively evaluating pulmonary venous return anomalies, atrioventricular septal defects, and valve abnormalities using multiple modalities, such as TEE or multidetector CT, is essential.

## Conclusion

Robotic repair can serve as a viable and therapeutically effective alternative for cases of CS-ASD with concomitant tricuspid annuloplasty.

## Supplementary Information

Supplementary Material 1. Robotic patch repair of a coronary sinus atrial septal defect

## Data Availability

The datasets used and/or analyzed during the current study are available from the corresponding author on reasonable request.

## Abbreviations

CS-ASD: Coronary sinus atrial septal defect
ASD: Atrial septal defect
CS: Coronary sinus
PLSVC: Persistent left superior vena cava
TTE: Transthoracic echocardiography
TEE: Transesophageal echocardiography
CT: Computed tomography
